# Estimation of reading subjective understanding based on eye gaze analysis

**DOI:** 10.1371/journal.pone.0206213

**Published:** 2018-10-25

**Authors:** Charles Lima Sanches, Olivier Augereau, Koichi Kise

**Affiliations:** Osaka Prefecture University, Sakai, Osaka, Japan; Swansea University, UNITED KINGDOM

## Abstract

The integration of ubiquitous technologies in the field of education has considerably enhanced our way of learning. Such technologies enable students to get a gradual feedback about their performance and to provide adapted learning materials. It is particularly important in the domain of foreign language learning which requires intense daily practice. One of the main inputs of adaptive learning systems is the user’s understanding of a reading material. The reader’s understanding can be divided into two parts: the objective understanding and the subjective understanding. The objective understanding can be measured by comprehension questions about the content of the text. The subjective understanding is the reader’s perception of his own understanding. The subjective understanding plays an important role in the reader’s motivation, self-esteem and confidence. However, its automatic estimation remains a challenging task. This paper is one of the first to propose a method to estimate the subjective understanding. We show that using the eye gaze to predict the subjective understanding improves the estimation by 13% as compared to using comprehension questions.

## Introduction

The recent development of small and inexpensive devices has led to a breakthrough in the education field. It allows students to learn from anywhere at any time and their learning activity can be recorded and analyzed. Such analysis is used to enhance their learning experience and provide new services such as context-aware learning systems [1] or adaptive teaching systems [2]. Moreover, the learning activity of a student can be used by a teacher as a feedback of the lesson comprehension [3], or by other students as a point of comparison. The idea is to set an environment where the students can be in a learning situation at any moments even if they are not aware of it. Such environment is called a “Ubiquitous Learning Environment” [4].

Ubiquitous learning environments have been widely explored in foreign language learning [5, 6] which demands a life-long practice to develop writing, listening, speaking and reading skills. Among these skills, reading has been proven to be a key activity [7] since it helps not only the development of conversation skills but also writing skills [8].

We distinguish two types of understanding: the objective understanding and the subjective understanding. The objective understanding can be measured by answering comprehension questions about the content of the text. It is evaluated in most language proficiency tests such as the TOEIC [9]. The subjective understanding measures the reader’s perception about his own understanding. It is linked to the reader’s confidence, self-esteem or motivation [10, 11]. Thanks to the subjective understanding it is possible to detect when the reader thinks he understood the content of the text but actually did not. Usually, the subjective understanding is estimated by asking the reader to rate his own understanding. However, this type of evaluation is not compatible with a ubiquitous learning environment where an automatic estimation is preferable.

Researchers have shown that the understanding of a text is related to our eye movements while reading [12–14]. More generally, it has been proved that the reading behavior is correlated to the eye movements patterns [15–17]. Therefore, we make the hypothesis that it is possible to use the relation between understanding and eye movements to predict the text subjective understanding. Moreover, the recent development of affordable eye tracking technologies makes it possible to use it in an everyday life situation. Previous work using eye tracking technologies could only be made in laboratory settings with devices costing up to 25,000 USD, but nowadays some devices cost around 150 USD which make them usable in everyday life.

Our research goal is to use the eye tracking technology to build a system which automatically estimates the reader’s subjective understanding. As a first step toward this goal, we propose in this paper a method to automatically estimate the overall subjective understanding of several Japanese texts based on eye tracking.

We show that we obtain 13% improvement in the estimation of the overall subjective understanding by using the eye gaze as compared to using comprehension questions. Moreover, because we aim to predict the understanding for each text independently, we analyze the effect of reducing the number of read texts on our algorithm. We show that not only analyzing the eye gaze is always more effective than using the comprehension questions, but also the smaller the number of read texts, the greater is the performance difference between both methods.

The contributions of the paper are twofold:

1. we provide a method to estimate subjective Japanese text understanding based on the eye gaze,
2. we show that the analysis of the eye gaze can predict the user’s subjective understanding more efficiently than by analyzing the comprehension question answers.

The remainder of the paper is organized as follows. First, we present some related work on eye gaze while reading, and document understanding using eye movements. Next, we explain our algorithm and in particular which information from the eye gaze we use to deduce the subjective comprehension of a text. Then we present the database, the readers and the detailed results for our experiment. Finally, we draw a conclusion and discuss the future work.

## 1 Related work

The eye movements while reading is composed of two categories: saccades and fixations. A fixation is a short stop on a part of a word during reading which lasts about 250 *ms* and a saccade corresponds to the quick movement of the eyes between two fixations.

Rayner has shown that these two features of the eye movements pattern change in accordance with the comprehension of the reader [12]. For example, the number of fixations is increased if the reader does not understand the text he is reading. Also, the readers tend to produce more backward saccades (rereading regression) if they have difficulty to understand the text.

Kunze *et al*. have also tried to show that there is a link between the reading pattern and the reading skills of the reader [18]. In particular, they tried to show that a reader tends to focus on a smaller number of words in the text if they have higher English reading comprehension skills. However, not only their database was small (5 participants reading 5 texts), but they also concluded that they did not find a statistically significant correlation between eye gaze and text understanding.

In their study Yoshimura *et al*. have tried to classify the English skill of readers in 3 classes: low, middle and high using eye gaze features [19]. The readers are assigned to a class depending on their TOEIC score which is a classic question-based language assessment test. As a consequence, the subjective understanding is not estimated in this study.

As an improvement of Yoshimura *et al*.’s work, Augereau *et al*. have proposed a method to estimate the TOEIC score of a student using the eye gaze information [20]. TOEIC is a test for assessing English proficiency. It includes a reading part and a listening part and the examinee is given a score, ranging from 10 to 990 points, which represents his English comprehension. Since the global TOEIC score and the reading part score are highly correlated [21], they have tried to estimate the TOEIC score using only the reading part. Each participant has been asked to read a text extracted from TOEIC test and to answer the questions related to it, while their eye movements were recorded. However, in order to predict the score, they use the correctness of the answers as an important feature. The estimation cannot be done without asking questions about the text to the participants. Moreover, no information about the participant’s estimation of his own understanding has been asked during the experiment. Therefore, only the objective understanding is evaluated in this study.

Karolus *et al*. have proposed a method to detect whether a displayed sentence is in a language understandable to the reader [22]. In particular, they have shown that fixation and blink durations are impacted by the language proficiency of the reader. However, this study has been conducted by only analyzing the understanding of very simple questions such as “How many days are within a week?”, on a very short period of time (4.5 seconds). Since the questions are very basic, the sentence difficulty is not taken into account in the study and could impact the reading behavior. Therefore, the degree of understanding of the sentence cannot be estimated.

Martinez-Gomez *et al*. have also proposed a method to recognize the language skill of a reader using eye gaze features [23]. Their method has been designed to retrieve both the subjective and objective understandings of the reader. The objective understanding, defined as the TOEIC or TOEFL score of the participants can be correctly estimated by their method. However, the reader’s perception of their own English ability, defined as a self-reported measure (beginner, intermediate or advanced) cannot be retrieved by their algorithm. They have shown there is a considerable difference in the objective measure of the understanding, and the subjective participants’ perception of their own ability.

The related work shows that it is possible to get some information about the comprehension of the reader by analyzing his eye movements. Moreover, estimating an objective measure of the language ability like the TOEIC score can be performed with a good accuracy. However, no work has been done for predicting the subjective understanding. In the following, we propose a method to predict the subjective understanding of Japanese texts by analyzing the eye movements of the reader.

This work is the continuation of the pre-study presented in [24]. It contains new results and an extended analysis.

## 2 From the understanding to the eye gaze

In this section, we describe the differences between objective and subjective understanding introduced in the Introduction section. In particular, we explain how important is the subjective understanding in the context of education. We then describe the features which can be extracted from the reading pattern to predict the subjective understanding.

### 2.1 Subjective and objective understanding

The student’s understanding of some reading material can be divided into two categories: the objective understanding and the subjective understanding. The objective understanding can be measured by using comprehension questions. It is usually measured via a score (the number of correctly answered questions) and is independent of the perception of the reader on his own understanding. In this study, we measure a different kind of understanding: the subjective understanding. The subjective understanding is measuring how well the student thinks he understood a text. The perception of a student on his own understanding is a key factor which will modify his self-esteem, his self-confidence, and his motivation [10, 11]. Moreover, it is an important information for the teacher (or the system, in a fully automated environment) who can adapt his teaching strategy according to it. For a student who answers a comprehension question, different scenarios can occur:

1. The student answered correctly to the comprehension question and he thinks that he understood the text
2. The student answered incorrectly to the comprehension question and he thinks that he did not understand the text
3. The student answered correctly to the comprehension question but he thinks that he did not understand the text
4. The student answered incorrectly to the comprehension question but he thinks that he understood the text

Although scenarios 1 and 2 are trivial, scenarios 3 and 4 require more attention from the teacher. These scenarios are accessible only if we can measure the reader’s subjective understanding.

In our study, we want to focus on the subjective understanding prediction. We compare the performance of two estimators to estimate the reader’s subjective understanding of one text: the correctness of a comprehension question and the eye gaze pattern of the reader. Objective and subjective understanding are different but correlated, and comprehension questions are the most common way to estimate the students’ objective understanding. Therefore, it makes sense to try to use it as an estimator of the subjective understanding. Moreover, the texts of our study are extracted from the Japanese Language Proficiency Test, a standardized test to assess one’s Japanese language ability. For each text, the associated comprehension question has been carefully designed by the test organizers to assess the student’s objective understanding of the text. But our hypothesis is that the reader’s eye gaze can be used to estimate the subjective understanding, and is a better estimator than the correctness of comprehension questions answers.

### 2.2 Features analysis

**Fig 1:**
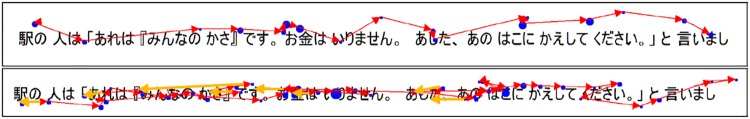
Eye gaze of 2 readers reading the same text. The fixations are represented by a blue dot which diameter depends on the duration: the more the reader looked at a specific region of the text, the bigger the dot is. The saccades between 2 fixations are represented by red strokes which direction is indicated by the arrow. The backward saccades (regressions) are represented in yellow. The first reader (upper part) understood the content of the text and the second did not understand the content of the text.

As a starting point of the analysis let’s compare the recording of a reader who did not understand what he has just read and a reader who almost completely understood the text he read. Fig 1 illustrates these two different scenarios. If we compare the saccades and the fixations of the two recordings we can find several differences: direction and length differences for the saccades, numbers and position differences for the fixations. These differences illustrate that the information of the understanding is included in the fixations and saccades pattern. Moreover, according to the literature, the reader’s fixations duration depends on his understanding [25]. Then, by analyzing several examples we can formulate hypotheses for two categories of readers:

1. Readers who understand the reading material:
  - **H1**: The reader will produce few, spread and quick fixations.
  - **H2**: The saccades produced by the reader are long and there is almost no backward saccades.
2. Readers who do not understand the reading material:
  - **H3**: The reader will produce numerous, grouped and long fixations.
  - **H4**: The saccades produced by the reader are short with many backward saccades.

Therefore, to estimate the understanding we can look for features related to fixations or related to saccades.

#### 2.2.1 Fixations and saccades features

According to the hypotheses **H1** and **H3**, the more the reader understands the text the less fixation he will produce. As a consequence, **f1** *the number of fixations* is a good candidate as a feature to predict the understanding.

According to the hypothesis **H2**, a reader who understands the text will produce long forward saccades and a few numbers of backward saccades. Conversely, according to the hypothesis **H4** a reader who did not understand the reading material will produce long saccades and many forward saccades. We can select **f2** *the number of forward saccades*, **f3** *the number of backward saccades* to estimate the understanding. To know how fast the reader is reading we use the idea of “saccade velocity” which is defined as:

$$\begin{array}{c} s_{v}=\frac{s_{l}}{s_{d}} \end{array}$$ (1)

with *s*_*v*_, *s*_*l*_, *s*_*d*_ the saccade velocity, the saccade length and the saccade duration respectively. We can use **f4** *the standard deviation of saccades velocities* as features to reflect the reading speed.

### 2.3 Features normalization

Some of the features previously described are highly dependent on the text length. For example, it is normal to produce more fixations on a long text because it contains more characters (or words). In the same way, it is normal to produce more forward saccades on a long text than a short text. Then, we can obtain variability on these features values because of the length of the text and not because of differences in the understanding. Therefore, it is essential to normalize the features in order to be independent from the length of the text. In order to do it, some features are normalized by the number of characters in the text and for other features, we use the standard deviation over all the recording. The normalization is performed for each text.

The features relevant to estimate the subjective reading understanding, the corresponding normalizations, and the associated hypotheses are gathered in Table 1.

**Table 1:** Features used to determine the reader comprehension of a text with their corresponding normalization.

Feature number	Feature	Normalization	Hypothesis
1	Number of fixations	Number of characters	H1,H3
2	Number of forward saccades	Number of characters	H2,H4
3	Number of backward saccades	Number of characters	H2,H4
4	Standard deviation of saccades velocities	Not needed	H2,H4

### 2.4 Regression

Once we have determined which features contain the subjective understanding information, we can see the retrieval of the reader’s comprehension as a minimization problem. Let *V* be the vector we obtain after extracting the *n* features from the raw eye gaze: *V* = [*f*_1_ *f*_2_ … *f*_*n*_] with *f*_*n*_ the value of the feature *n*. And if we call *U* the reader’s subjective understanding and *U*_*E*_ the corresponding estimated subjective understanding. We then look for a function *F*(*V*) which minimizes the distance |*U* − *U*_*E*_|.

To evaluate the performance of our algorithm, we can compute the average mean distance over all participants. If we call *N* the number of participants, the performance is computed as *E*, the mean absolute error of the understanding estimation:

$$\begin{array}{c} E=\frac{1}{N}\sum_{i=1}^{N}\left|U-U_{E}\right| \end{array}$$ (2)

### 2.5 Global average understanding

#### 2.5.1 From the eye gaze

In our method, we compute the average subjective understanding of all the read texts for each user and try to predict this global average subjective understanding using the features extracted from the eye gaze. Because the average subjective understanding over all the texts read by a reader is a continuous value, our problem is not a classification problem. As a consequence, we use a Support Vector Regression (SVR) method to estimate this average subjective understanding. In order to find the global subjective understanding over all the texts read by each user, we compute the average of features over all the texts. Let *V*_1_ = [*f*_11_ *f*_12_ … *f*_1*n*_] be the vector we obtain after extracting the *n* features from the raw eye gaze of the text 1. In the same way we have *V*_2_ = [*f*_2*n*_ *f*_2*n*_ … *f*_2*n*_] the vectors computed from the text 2. We can build *V*_1_,…, *V*_*p*_ the *p* vectors computed from the *p* texts read by the user. Then we will build a vector *X* = [*a*_1_ *a*_2_ … *a*_*n*_] with

$$\begin{array}{c} a_{y}=\frac{f_{1y}+f_{2y}+f_{3y}+\ldots +f_{py}}{p} \end{array}$$ (3)

This vector *X* will be the input of our SVR.

**Fig 2:**
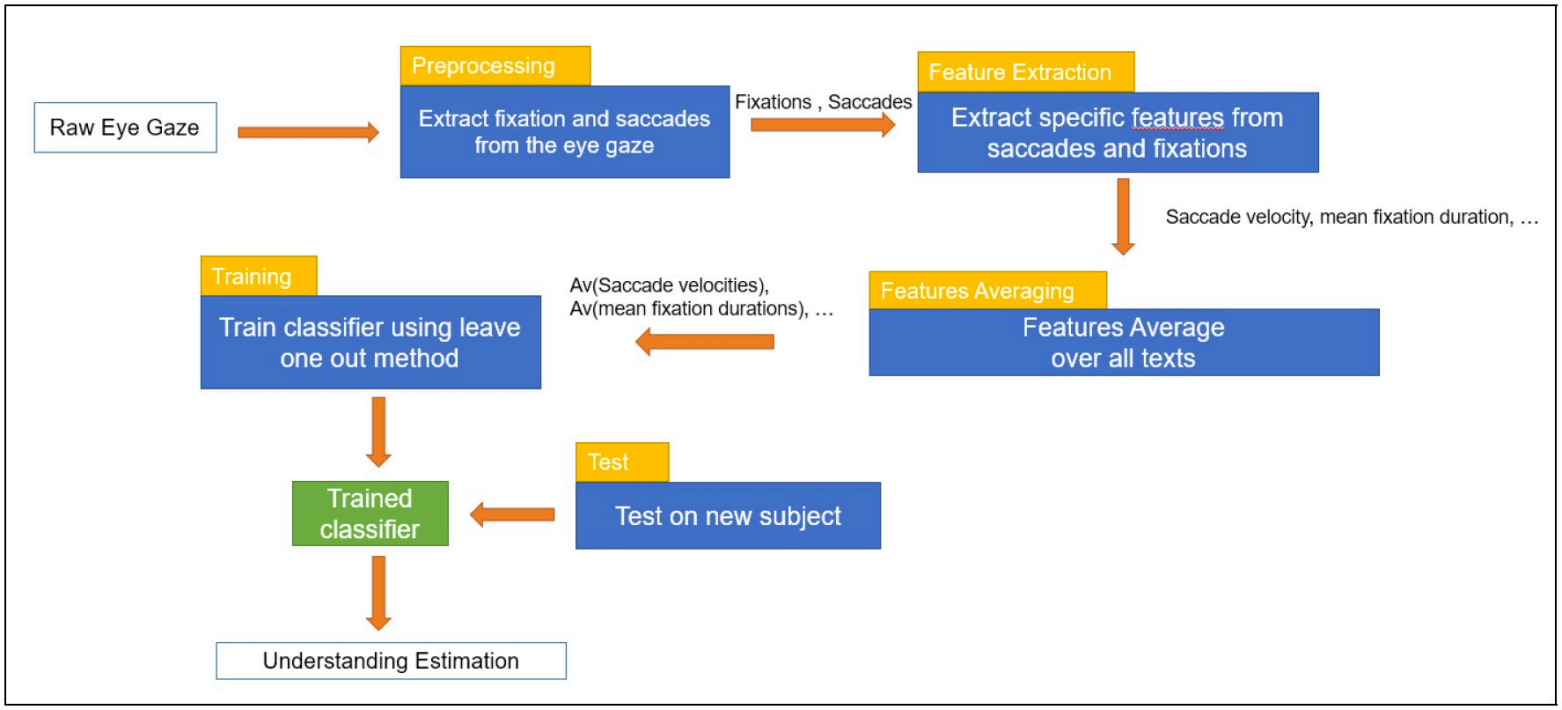
Algorithm overview from the raw eye gaze to the prediction of the understanding.

The overview of the algorithm is described in Fig 2. First, we extract the fixations and the saccades from the eye gaze, then we extract some features from it. We average the features for each text read by the user and use these average features as an input for the SVR. We train our model using a leave-one-participant-out strategy. Therefore, our method is participant independent.

#### 2.5.2 From the answers

In order to evaluate the subjective understanding estimated by using the eye gaze features, we compare it with the same estimation using the correctness of the answers given by the participant. The questions are text comprehension questions directly extracted from JLPT textbooks. The answer (*wrong* = 0 or *right* = 1) is used as a feature for the classifier. The answers to all the questions answered by one participant are summed and the sum is divided by the number of questions answered by the participant. The result is an average score *S* which will be the input of the classifier.

## 3 Experiment

This study has been approved by the ethics committee of the graduate school of engineering, Osaka Prefecture University. The authors received a written consent from all the participants. The participants have been recruited in February 2016 via e-mail or verbal invitation. All the participants were university students aged between 20 and 28 years old. All the data were anonymised.

In this section, we describe the experiment protocol. The eye tracker is used in this experiment is a nonprofessional Tobii eye tracker (http://www.tobii.com/). With an accuracy <0.6°, a precision <0.25°, and a latency <50ms, such eye trackers can be used to successfully measure fixations and saccades [26]. A research license is required to use it for research purposes. This kind of eye tracker is inexpensive and therefore can be spread to a large community. The eye tracker is attached to a screen and the documents are displayed on this screen. The head of the reader needs to be approximately between 45 to 80 cm apart from the screen. The reader is free to move his head, no bite bar nor head fixation have been used in order to be close to real reading conditions. At 70 cm distance, the allowed head movement is a plane of approximately 48 x 39 cm. If the participant moves more, the eye gaze will not be detected anymore by the eye tracker.

**Fig 3:**
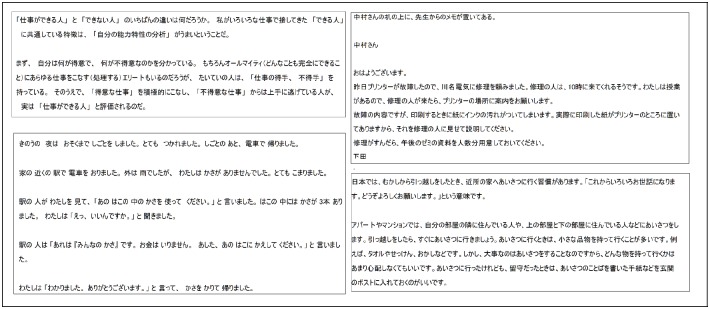
Example of JLPT texts. Level N2 (top left), level N3 (top left), level N4 (bottom right), level N5 (bottom left).

17 participants (7 females, 10 males) were asked to read 19 Japanese texts while their eye movements were recorded. The participants are from different nationalities: 4 Chinese, 10 French, 1 German, 1 Taiwanese and 1 Vietnamese. They are all university students (from 20 years old to 28 years old), with different majors and Japanese abilities. The texts are extracted from some Japanese Language Proficiency Test textbooks and correspond to the different levels of the JLPT: from N2 to N5, N5 being the easiest. Because of the lack of people capable of understanding the N1 level (most difficult level) we did not use any N1 level texts in this experiment. Samples of the used texts are shown in Fig 3. Each participant had to read four texts N2, five texts N3, five texts N4, five texts N5. The average number of characters per text is 210. The texts were written using the font MS UI gothic, and the font size was 30 pt. For each text, the participant was asked to answer one comprehension question designed by the test organizers.

Then, the participant was asked to rate his understanding of each text from 1 to 5. Such self-assessed methods induce bias in the measures which can be caused by four factors [27]:

- Failure in memory: we tried to minimize this factor by asking the participant to rate his understanding immediately after reading the text.
- Lack of appropriate motivation: We minimized this factor by paying the participants for the experiment.
- Failure in communication or lack of knowledge: We minimized this factor by explaining carefully which marks corresponds to which level of understanding (equivalence).

For each value of the subjective understanding, the corresponding equivalence has been given.

- 1: Aborted the text
- 2: Understood almost nothing
- 3: Understood part of the content
- 4: Understood the content but some parts were unclear
- 5: Understood everything

In the evaluation of our algorithm, we did not take into account the case 1 as the participant did not read a large part of the corresponding text. Then, the average understanding we want to predict is a continuous value within the interval [2;5]. The distribution of each recording in the database is shown in Table 2. The descriptive statistics of eye movements data for this experiment is shown in Table 3. The data used to compute the statistics is publicly available (https://github.com/chalulu/Subjective-Understanding).

**Table 2:** Understanding distribution in the database.

Understanding	Number of Recordings
1	23
2	45
3	23
4	74
5	137

**Table 3:** Descriptive statistics of eye movements data (average and standard deviation for each feature).

Eye Movements Data	Statistics	
	*Average*	*STD*
Fixation count	111.53	65.89
Fixation duration (ms)	291.48	70.11
Forward saccade count	82.13	49.17
Backward saccade count	28.38	19.43
Ratio forward/backward saccade	3.15	1.38
Saccade velocity (px/ms)	0.32	0.10
Forward saccade length (px)	114.99	39.64
Backward saccade length (px)	-327.46	128.86

### 3.1 Results and analysis

This section gathers the results of our experiment. As a first analysis of the results, we compute the average value of the features for the different subjective understanding values. Table 4 shows that for most of the features, there is a correlation between the value of the feature and the self-assessed understanding. The p-values were also computed using the Pearson method to show the significance of this correlation. Since the feature **2** did not show a strong significance of the correlation between its value and the self-assessed understanding, the model was capable of finding a non-linear relationship between the two values.

**Table 4:** Average features values for the different subjective understandings. The p-values were computed to show the significance of the correlation between the subjective understanding and the features value.

Subjective Understanding	Feature Number (see Table 1)			
	1	2	3	4
2	0.74	0.45	0.08	0.20
3	0.71	0.40	0.09	0.18
4	0.61	0.46	0.11	0.16
5	0.46	0.49	0.14	0.12
p-value	1.10^−12^	7.10^−3^	2.10^−9^	1.10^−7^

The statistical method used here assumes that the data is normally distributed. The normality has been assumed after examination of the Quantile-Quantile (QQ) plots for each feature. Because of the number and the size of the plots, they have not been included in the paper. The QQ plots and corresponding computed values have been made publicly available (https://github.com/chalulu/Subjective-Understanding).

For our main result, we compare the precision of the estimation using the eye gaze features with the one using the answer feature. We want to know which estimation is the closest to the real average understanding value in the interval [2;5]. The corresponding results are shown in Table 5. In our experiment, we did not use the recordings corresponding to aborted texts. Therefore, the number of texts is not the same for all the participants.

**Table 5:** Error in the understanding estimation using eye gaze features or the answer feature.

Features	Mean absolute error
Eye Gaze	0.26
Answers	0.30

**Fig 4:**
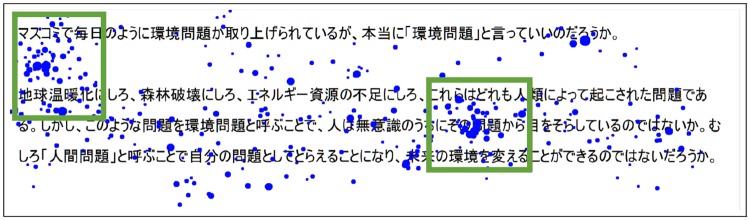
Example of recording: The reader struggled with two parts of the text but read the rest more smoothly. The understanding information of these two parts of the text is included in the eye gaze but not in the question.

In this table, we can see that using the eye gaze to predict the subjective understanding is more effective than using the correctness of the answers. This can be explained because the answers to the questions cannot relate perfectly to the general comprehension of a text. For example, in Fig 4 we can see that the reader struggled with two specific parts of the text (green squares): the fixations are condensed and the saccades are very short. Therefore, the eye gaze indicates which parts were difficult for the reader. However, the reader will be able to answer correctly if the information given in the difficult parts is irrelevant for the right answer. The information of the understanding is not accessible by using the question because it cannot cover all the text parts. In order to be more efficient, it would be necessary to ask questions about all the parts of the text, which is impossible. Conversely, the understanding of every part of the text is included in the eye gaze and then the global understanding is easier to predict.

**Fig 5:**
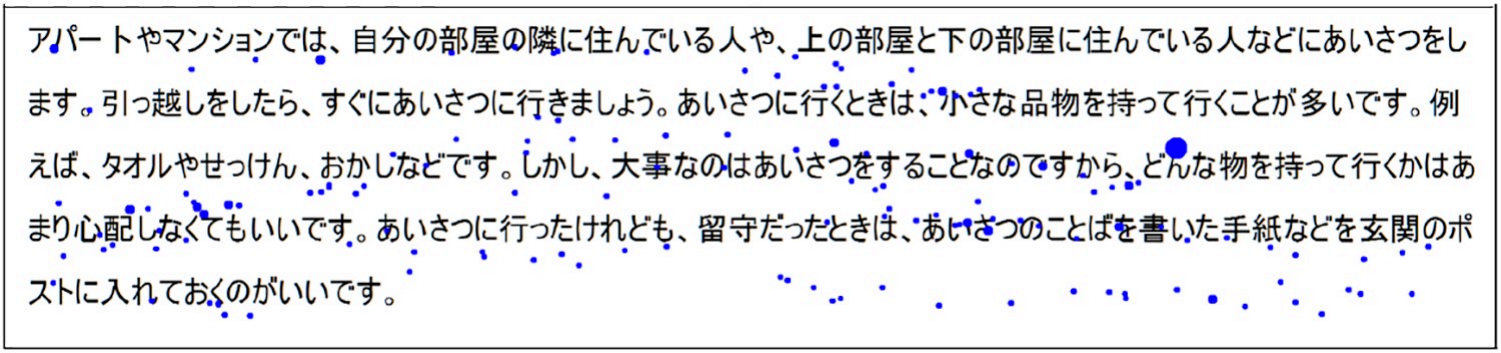
Example of recording where the reader keeps reading smoothly: There is no fixation agglomeration and the fixation duration are small (dot diameter). However, the reader did not understand the text very well and gave a wrong answer for the comprehension question.

However, the algorithm can be “fooled” by the reader’s reading behavior. For example, if the reader keeps reading smoothly even if he does not understand the text. In that case, the lack of understanding will not be included in the eye gaze. This case is represented in Fig 5. There is no fixation agglomeration in this recording and the small fixation duration indicates that the reader read smoothly the text. However, he did not answer correctly the comprehension question, so he clearly misunderstood some part or all the text. This can explain that answering the question can be more accurate than analyzing the eye gaze for some specific reading behaviors. A solution to correct this error will be discussed in the section 4.

**Fig 6:**
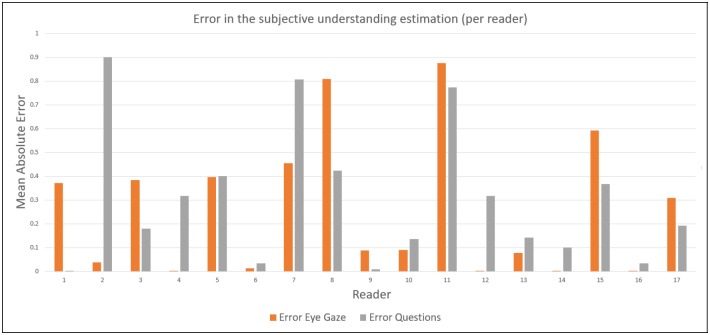
Mean absolute error in the understanding estimation using two different types of information: The eye gaze or the comprehension question.

The comparison of the mean absolute error participant by participant between the answer features and the eye gaze features is shown in Fig 6. In this figure, we can see that using the eye gaze is globally more effective than using the comprehension question, but for some specific reading behavior (for example reader 1) answering the question is more efficient than analyzing the eye gaze.

### 3.2 Impact of the number of texts on the understanding estimation

After analyzing the recording of 19 texts read by a user and estimating the global understanding of these texts, we analyze the impact of reducing the number of texts on the algorithm performance. Less information will be available to predict the understanding if we reduce the number of texts, which makes the estimation more difficult. The estimation is then more and more difficult. In the limit case, we try to estimate the understanding for each text individually. In this limit case, we cannot compare the performance of our eye gaze method with the question feature method because only one question has been asked for each text. The correctness of the answer is a binary value, and this information is not precise enough to predict an understanding which ranges in the interval [2;5].

**Fig 7:**
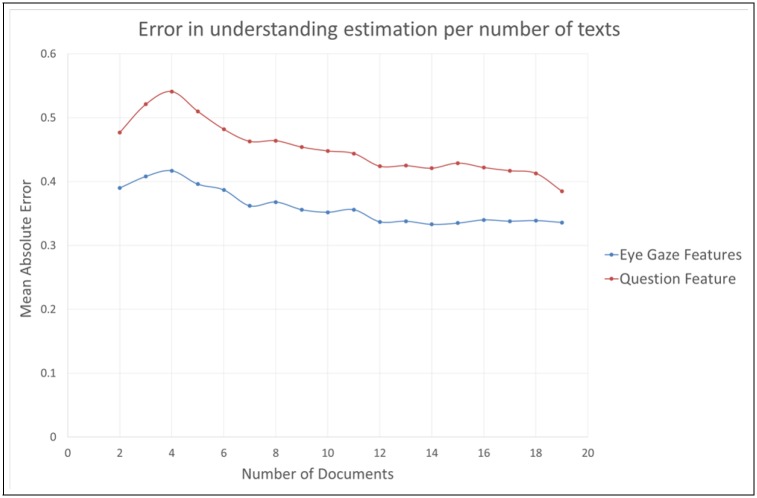
Error in the understanding estimation per number of texts. The eye gaze features method is more accurate than the comprehension question method for any number of documents. For a large number of documents (>15) the performance of both method tends to be similar.

**Fig 8:**
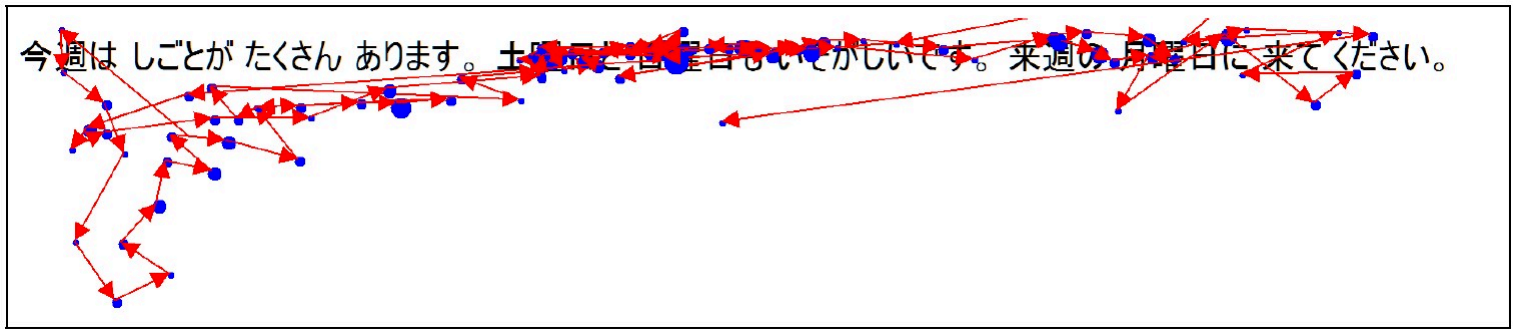
Unusual reading behavior: Even if the participant perfectly understood the text, he reread and focused a lot on some parts of the text.

The results are gathered in Fig 7. Because we removed the aborted texts, all participants have not read the same number of documents. As a consequence for some participants, the number of available texts is smaller than the number of texts tested. When the number of texts tested is greater than the number of texts available for a participant, we still include this participant in our computation by using all the texts he read. We can see that the fewer number of texts, the lower the precision for both the eye gaze features method and the comprehension question method. This can be explained because for a fewer number of texts the algorithm is more sensitive to unusual reading behaviors. For example, Fig 8 shows a recording with numerous backward saccades and fixations. This means the participant reread a lot some parts of the text and focused on some words. Therefore, the algorithm estimated that he did not understand the text so well (estimated understanding = 2.6). However, when asked about his understanding, the participant answered 5, which corresponds to a complete comprehension of the text. This behavior can occur when the participant reread some parts of the text to check again his own understanding. Knowing that the participant will be asked one comprehension question, he can be tempted to check again the text in order to be sure he will be able to answer the question.

We also notice that analyzing the eye gaze is more accurate than using the comprehension question for any number of documents. However, for a number of texts ranging from 15 to 19, the comprehension question method tends to improve its accuracy although the eye gaze method performance is stable. This indicates that for a large number of texts and a large number of questions, there is as much information brought by the question as information brought by the eye gaze.

## 4 Discussion on the sources of error and limitations

In the results above, we have shown that using the eye gaze is an efficient way to predict the understanding of a text for a participant. Analyzing the fixations and the saccades information helps to distinguish a reader who did not understand the text from a reader who did understand, confirming our hypotheses **H1**, **H2**, **H3** and **H4**. However, we have underlined some limitations which affect the algorithm performance. One of the first error is linked to the eye tracker accuracy. Because of the vertical error of the eye tracker, some fixations are not on the text characters. However, because we do not use the position of the fixations relatively to the text in our algorithm (only the fixations number and their duration), this misplacement does not affect the algorithm. As a consequence, no fixations were excluded from the analysis. Nevertheless, the recordings can contain some noise which will be interpreted as a specific reading behavior by the algorithm. For example, in the Fig 8 we can see a drift at the beginning of the recording which is an error of the eye tracker. Moreover, with the vertical error of the eye tracker, some fixations are not on the text characters. This kind of errors can be avoided by improving the calibration step or by using post-processing algorithms [28].

Another source of errors can be unusual reading behaviors. If the reader reads the text smoothly although he did not understand it, or if he rereads many parts of the texts although he perfectly understood them, the algorithm will not be able to estimate accurately the reading understanding. This could be detected by using the content of the text as an additional feature: studies have shown that the word length and the visual complexity can affect the reading behavior of Japanese sentences even for native speakers [29]. In particular, the visual complexity of Japanese characters (made from more or fewer strokes) directly influences the reading time and the text areas fixed by the reader. Therefore, in a text containing areas of simple complexity and difficult complexity, the reading behavior of the reader should change. If it does not change because the reader read smoothly forward the text, it can be detected as an unusual reading behavior and processed differently. It would also be possible to compare the reading behaviors of participants with similar Japanese skill to detect an unusual reading behavior. However, this would require to know the Japanese skill of the reader in advance. This solution cannot be used in the context of a test but can be considered in the context of a self-understanding quantification. Also, in the context of our study, recording only people with similar profiles might lead to similar subjective understanding and the algorithm would not generalize well. Therefore, we recruited participants from various nationalities and various Japanese reading abilities since this variation helps the algorithm to generalize with different readers’ profiles.

Our study is limited to the analysis of the reader’s subjective understanding. Since no questions have been asked about it, no conclusion can be drawn about the reading ease or reading fluency. Similarly, we use the comprehension questions as a point of comparison to evaluate our eye gaze based method but they are not used by the algorithm. Therefore, the objective understanding cannot be directly predicted from this method. The prediction of the reader’s objective understanding with the eye gaze has been studied in the past and requires the extraction of different eye gaze features [30].

## 5 Conclusion

In this paper, we presented a method to estimate the reading subjective understanding based on the eye gaze. Our method is based on the analysis of average features to predict a subjective understanding after reading several short texts. We showed that our method is more accurate than asking questions about the texts. With our method, the mean absolute error is 0.33 against 0.38 by using the comprehension question.

We have also shown that even if we reduce the number of texts, analyzing the eye gaze is still more efficient than using the comprehension question. Moreover, the smaller the number of read documents, the greater is the difference in performance. However, there are still some errors in the subjective understanding estimation. This can be explained by the small size of the texts and by some specific reading behaviors such as repeated checking. We plan to use the content of the text, to detect unusual reading behavior which does not match with the visual complexity of the sentences.

Nevertheless, our method can be used in a ubiquitous learning environment as an input so that the learning material is automatically adjusted to the user’s subjective understanding. In the future, by using this understanding on texts of different levels, and by comparing with different users we plan to estimate the reader’s confidence about his Japanese ability. Also, because our method is not only meant to estimate the reader’s subjective understanding of only Japanese texts, we plan in the future to apply our method to other languages.
